# Ultra-deep targeted sequencing of advanced oral squamous cell carcinoma identifies a mutation-based prognostic gene signature

**DOI:** 10.18632/oncotarget.3768

**Published:** 2015-04-25

**Authors:** Shu-Jen Chen, Hsuan Liu, Chun-Ta Liao, Po-Jung Huang, Yi Huang, An Hsu, Petrus Tang, Yu-Sun Chang, Hua-Chien Chen, Tzu-Chen Yen

**Affiliations:** ^1^ Department of Biomedical Sciences, Chang Gung University, Taoyuan, 33302, Taiwan; ^2^ Genomic Core Laboratory, Chang Gung University, Taoyuan, 33302, Taiwan; ^3^ Graduate Institute of Biomedical Sciences, Chang Gung University, Taoyuan, 33302, Taiwan; ^4^ Department of Otorhinolaryngology, Chang Gung Memorial Hospital, Taoyuan, 33305, Taiwan; ^5^ Bioinformatics Core Laboratory, Chang Gung University, Taoyuan, 33305, Taiwan; ^6^ Department of Nuclear Medicine, Chang Gung Memorial Hospital, Taoyuan, 33305, Taiwan

**Keywords:** next-generation sequencing, oral squamous cell carcinoma, mutation, prognosis

## Abstract

**Background:**

Patients with advanced oral squamous cell carcinoma (OSCC) have heterogeneous outcomes that limit the implementation of tailored treatment options. Genetic markers for improved prognostic stratification are eagerly awaited.

**Methods:**

Herein, next-generation sequencing (NGS) was performed in 345 formalin-fixed paraffin-embedded (FFPE) samples obtained from advanced OSCC patients. Genetic mutations on the hotspot regions of 45 cancer-related genes were detected using an ultra-deep (>1000×) sequencing approach. Kaplan-Meier plots and Cox regression analyses were used to investigate the associations between the mutation status and disease-free survival (DFS).

**Results:**

We identified 1269 non-synonymous mutations in 276 OSCC samples. *TP53*, *PIK3CA*, *CDKN2A*, *HRAS* and *BRAF* were the most frequently mutated genes. Mutations in 14 genes were found to predict DFS. A mutation-based signature affecting ten genes (*HRAS, BRAF, FGFR3, SMAD4, KIT, PTEN, NOTCH1, AKT1, CTNNB1*, and *PTPN11*) was devised to predict DFS. Two different resampling methods were used to validate the prognostic value of the identified gene signature. Multivariate analysis demonstrated that presence of a mutated gene signature was an independent predictor of poorer DFS (*P* = 0.005).

**Conclusions:**

Genetic variants identified by NGS technology in FFPE samples are clinically useful to predict prognosis in advanced OSCC patients.

## INTRODUCTION

Oral squamous cell carcinoma (OSCC) is the most common malignancy of the oral cavity, accounting for more than 90% of all oral neoplasms [1]. Approximately 300,000 new oral cavity cancer cases and 145,000 oral cancer-related deaths were registered in 2012 (GLOBOCAN 2012, http://globocan.iarc.fr/), showing increasing trends from 2008 [2]. Risky oral habits (including smoking, alcohol drinking, and betel quid chewing) are major risk factors for OSCC development [2]. The 5-year overall survival (OS) and disease-free survival (DFS) rates for OSCC patients are approximately 70% and 67%, respectively [3, 4]. However, the prognosis of patients with advanced disease (stage III/IV) remains dismal [5–7]. Different prognostic factors have been identified in OSCC patients (e.g., tumor and nodal stages, tumor differentiation, AJCC stage, tumor invasiveness, treatment modalities, and surgical margins) [3, 7]. Although extracapsular spread (ECS) is generally considered as the main predictor of disease progression, alone it does not provide sufficient information on the patient's clinical course [7]. Approximately 36% of OSCC patients with ECS are able to achieve an acceptable survival, whereas poor OS rates can be observed in 40% of OSCC patients without ECS [6]. In this scenario, an improved molecular characterization of OSCC may be helpful for informing prognosis and devising tailored treatment strategies.

Mutations of the *TP53*, *CDKN2A*, *HRAS*, and *PIK3CA* genes have been frequently reported in OSCC samples [8, 9]. Moreover, mutations in *NOTCH1*, *FAT1*, and *CASP8* may promote the initiation and progression of OSCC [10–12]. However, the question as to whether such mutations may improve the prognostic stratification of OSCC remains open. Notably, previous studies mainly identified mutations in tumor suppressor genes (TSG) rather than oncogenes, the only exceptions being *HRAS a*nd *PIK3CA*. However, most small molecules and/or biological agents under clinical development can directly engage activating mutations in oncogenes [13, 14].

Herein, we sought to identify genetic alterations that may improve the prognostic stratification of advanced OSCC patients, being also actionable as potential treatment targets. To this end, we conducted ultra-deep sequencing of the mutational hotspot in 45 cancer-related genes using formalin-fixed paraffin-embedded (FFPE) primary tumor samples (*n* = 345). A total of 29 oncogenes and 16 tumor suppressor genes [TSG] were examined, including ABL1, AKT1, ALK, APC, ATM, BRAF, CDH1, CDKN2A, CSF1R, CTNNB1, EGFR, ERBB2, ERBB4, FBXW7, FGFR1, FGFR2, FGFR3, FLT3, GNAS, HNF1A, HRAS, IDH1, JAK3, KDR, KIT, KRAS, MET, MLH1, MPL, NOTCH1, NPM1, NRAS, PDGFRA, PIK3CA, PTEN, PTPN11, RB1, RET, SMAD4, SMARCB1, SMO, SRC, STK11, TP53, and VHL. The high-sequencing depth allowed a highly sensitive mutation detection and the availability of long-term follow-up data was useful for improving molecular prognostic stratification, ultimately favoring tailored treatment approaches.

## RESULTS

### Patient characteristics

**Figure 1:**
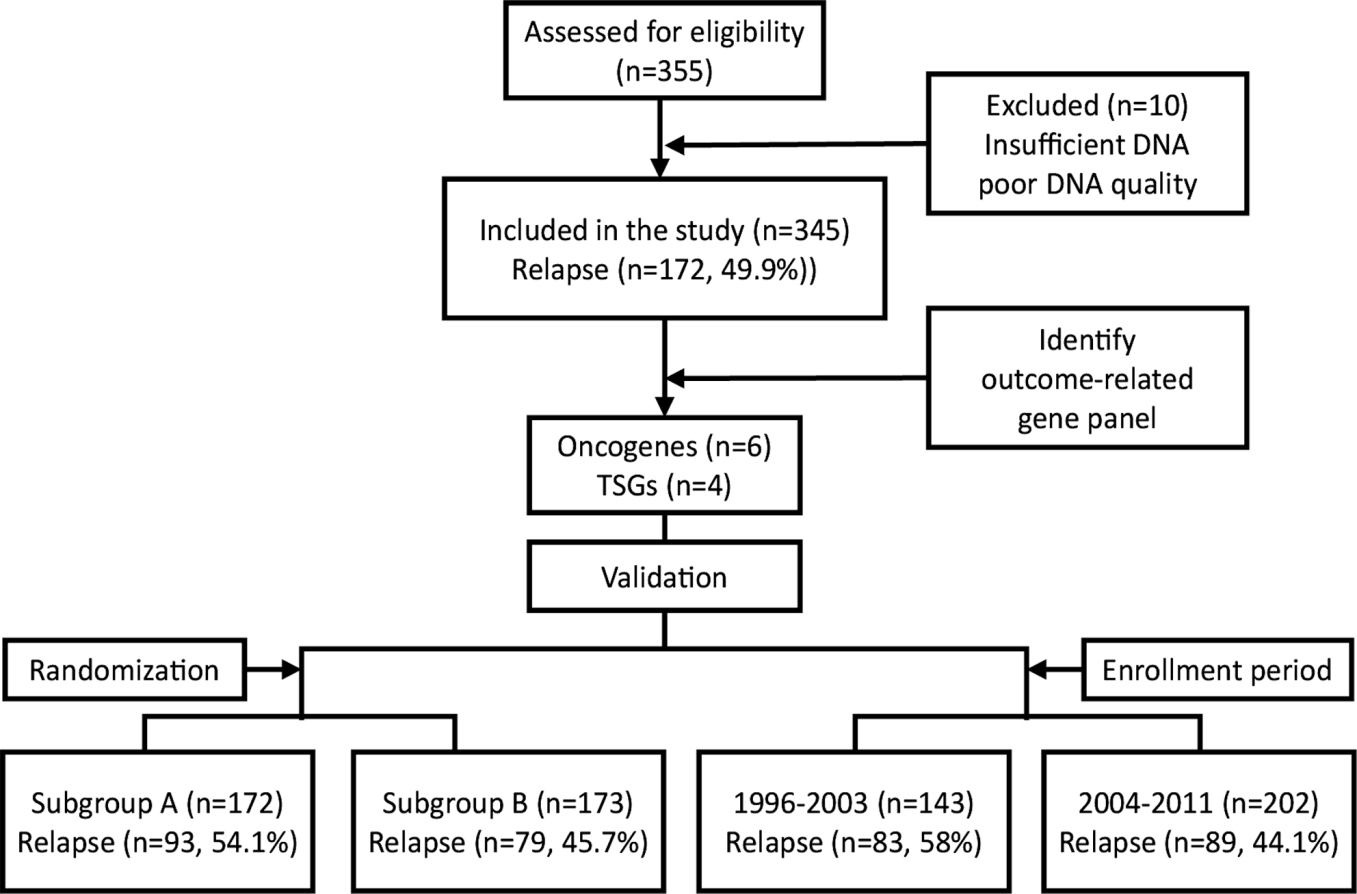
Selection of the study population

We obtained a total of 355 tumor samples from pN+ patients with previously untreated stage III/IV OSCC. NGS sequencing data were available for 345 patients (97.2%; 325 males and 20 females). Among the remaining 10 patients, 6 (1.7%) had insufficient DNA and 4 (1.1%) inadequate DNA quality (Figure 1). Tumor sites were as follows: bucca (*n* = 132, 38.3%), tongue (*n* = 130, 37.7%), alveolar ridge (*n* = 44, 12.8%), retromolar trigone (*n* = 16, 4.6%), mouth floor (*n* = 15, 4.3%), hard palate (*n* = 6, 1.7%), and lip (*n* = 2, 0.6%). The prevalence rates of pre-operative alcohol drinking, betel quid chewing, and cigarette smoking use were 71% (*n* = 246), 82% (*n* = 282), and 92% (*n* = 313), respectively. The pathological stage was p-stage III in 85 patients (25%) and p-stage IV in 260 patients (75%). After a minimum follow-up of 30 months (or censoring at the date of death), there were 172 cases (49.9%) of tumor relapse, with a median DFS of 65 months. A total of 201 patients (58.3%) had ECS. Table 1 summarizes the general characteristics of the study participants.

**Table 1:** Characteristics of OSCC patients in the entire cohort and subsets used for internal validation

Characteristics	Entire cohort	Randomization		Enrollment period	
		Subgroup A	Subgroup B	1996–2003	2004–2011
N (%)	345 (100)	172 (100)	173 (100)	143 (100)	202 (100)
Age at onset (years)					
< 65	307 (89.0)	157 (91.3)	150 (86.7)	132 (92.3)	175 (86.6)
≥ 65	38 (11.0)	15 (8.7)	23 (13.3)	11 (7.7)	27 (13.4)
Range	27 – 89	27 – 89	27 – 83	27 – 74	29 – 89
Mean ± SD	49.6 ± 11.0	49.3 ± 11.1	50.0 ± 11.9	48.3 ± 10.8	50.6 ± 10.9
Sex					
Male	325 (94.2)	166 (96.5)	159 (91.9)	136 (95.1)	189 (93.6)
Female	20 (5.8)	6 (3.5)	14 (8.1)	7 (4.9)	13 (6.4)
Alcohol drinking					
No	99 (28.7)	51 (29.7)	48 (27.7)	51 (35.7)	48 (23.8)
Yes	246 (71.3)	121 (70.3)	125 (72.3)	92 (64.3)	154 (76.2)
Betel quid chewing					
No	63 (18.3)	25 (14.5)	38 (22.0)	21 (14.7)	42 (20.8)
Yes	282 (81.7)	147 (85.5)	135 (78.0)	122 (85.3)	160 (79.2)
Cigarette smoking					
No	32 (9.3)	14 (8.1)	18 (10.4)	10 (7.0)	22 (10.9)
Yes	313 (90.7)	158 (91.9)	155 (89.6)	133 (93.0)	180 (89.1)
Pathological T status					
pT1–2	153 (44.4)	75 (43.6)	78 (45.1)	59 (41.3)	94 (46.5)
pT3–4	192 (55.6)	97 (56.4)	95 (54.9)	84 (58.7)	108 (53.5)
Pathological N status					
pN1	123 (35.7)	59 (34.3)	64 (37.0)	50 (35.0)	73 (36.1)
pN2	222 (64.3)	113 (65.7)	109 (63.0)	93 (65.0)	129 (63.9)
Pathological stage					
III	85 (24.6)	39 (22.7)	46 (26.6)	34 (23.8)	51 (25.2)
IV	260 (75.4)	133 (77.3)	127 (73.4)	109 (76.2)	151 (74.8)
Extracapsular spread					
No	144 (41.7)	68 (39.5)	76 (43.9)	53 (37.1)	91 (45.0)
Yes	201 (58.3)	104 (60.5)	97 (56.1)	90 (62.9)	111 (55.0)
Margin status					
≤4 mm	43 (12.5)	25 (14.5)	18 (10.4)	20 (14.0)	23 (11.4)
>4 mm	298 (86.4)	144 (83.7)	154 (89.0)	119 (83.2)	179 (88.6)
unknown	4 (1.2)	3 (1.8)	1 (0.6)	4 (100)	0 (0)
Treatment modality					
Surgery	25 (7.2)	12 (7.0)	13 (7.5)	10 (7.0)	15 (7.2)
Surgery + RT/CCRT	320 (92.8)	160 (93.0)	160 (92.5)	133 (93.0)	202 (92.8)
Relapse status					
No	173 (50.1)	79 (45.9)	94 (54.3)	60 (42.0)	113 (55.9)
Yes	172 (49.9)	93 (54.1)	79 (45.7)	83 (58.0)	89 (44.1)
5-yr survival rate (%)	50.8	46.3	54.7	43.2	56.4

Abbreviations: RT, radiotherapy; CCRT, concurrent chemoradiation.

### Sequencing results

We were able to achieve a 2410-fold mean sequence coverage for the targeted regions (97.5% of them were covered at >100 folds). The complete coverage details are provided in the Supplement (Data Supplement, Supplementary Table S1). A total of 1,269 non-synonymous (missense, nonsense, indel and splicing site) mutations with an allele frequency ≥ 3% were detected in 276 (80%) samples (Supplementary Figure S2). The average number of non-synonymous mutation per tumor was 4.6. However, the mutation rate was highly dependent on the specimen (from 1 to 166 mutations per sample). Sixty-nine samples (20%) had no detectable non-synonymous mutations. Based on the total number of mutations per tumor, the 276 specimens were divided into three different mutation groups, as follows: ultra-mutators (>50 mutations/tumor, *n* = 5), hyper-mutators (17−46 mutations/tumor, *n* = 7), and mutators (1−11 mutations/tumor, *n* = 265). The number of detected mutations was not significantly influenced by the sample storage time (Supplementary Figure S1), suggesting that even long-time stored FFPE specimens were suitable for NGS analysis. Using the remaining genomic DNA specimens, a total of 120 detected mutations were validated by means of Sanger sequencing or pyrosequencing. Of them, 99 (82.5%) were successfully validated (Data Supplement, Supplementary Table S2). The validation rate further increased to 96.1% (73 of 76) for genetic variants with an allele frequency >7.5%, suggesting that alternative sequencing methods may not be sufficiently sensitive for the detection of low-frequency mutations [15]. Missense mutations accounted for the majority (73.6%) of the identified variants, followed by nonsense mutations (14.6%). Insertions/deletions (indels) accounted for only 2.3% of all variants. We also analyzed the association between the number of mutations in each sample and risky oral habits (alcohol drinking, betel nut chewing, and cigarette smoking) (Supplementary Figure S3). The number of genetic mutations in tumor samples was significantly higher in smokers than in non-smokers (3.9 ± 14.8 vs. 1.6 ± 1.5, *P* = 0.0093) as well as in betel nut users compared with non-users (4.1 ± 15.5 vs. 1.9 ± 3.8, *P* = 0.0375). However, the mean number of sequence variants did not differ significantly in alcohol users compared with non-users (3.7 ± 16.8 vs. 4.37 ± 12.9, *P* = 0.9769).

### Mutational landscape in the targeted regions

**Figure 2:**
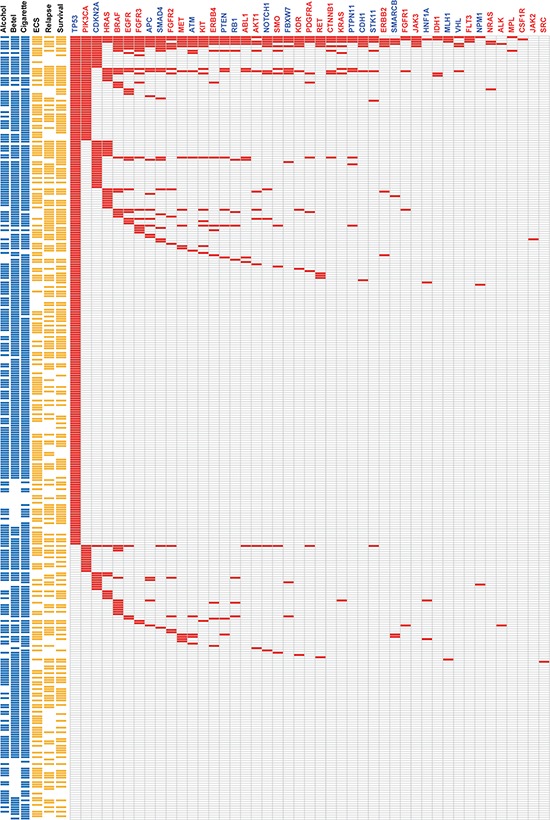
Heat-map representation of individual non-silent variants identified in 345 specimens (rows) (Left) Risk behaviors of individual patients. Individual highlighted in blue were exposed to alcohol drinking, betel nut chewing, and cigarette smoking, respectively. (Center) Clinical characteristics of individual patients. Individual highlighted in yellow were positive for ECS, had tumor relapse, or died. (Right) Mutation metrix for individual genes in each patient. Tumor suppressor genes are labeled in blue, whereas oncogenes are indicated in red.

**Figure 3:**
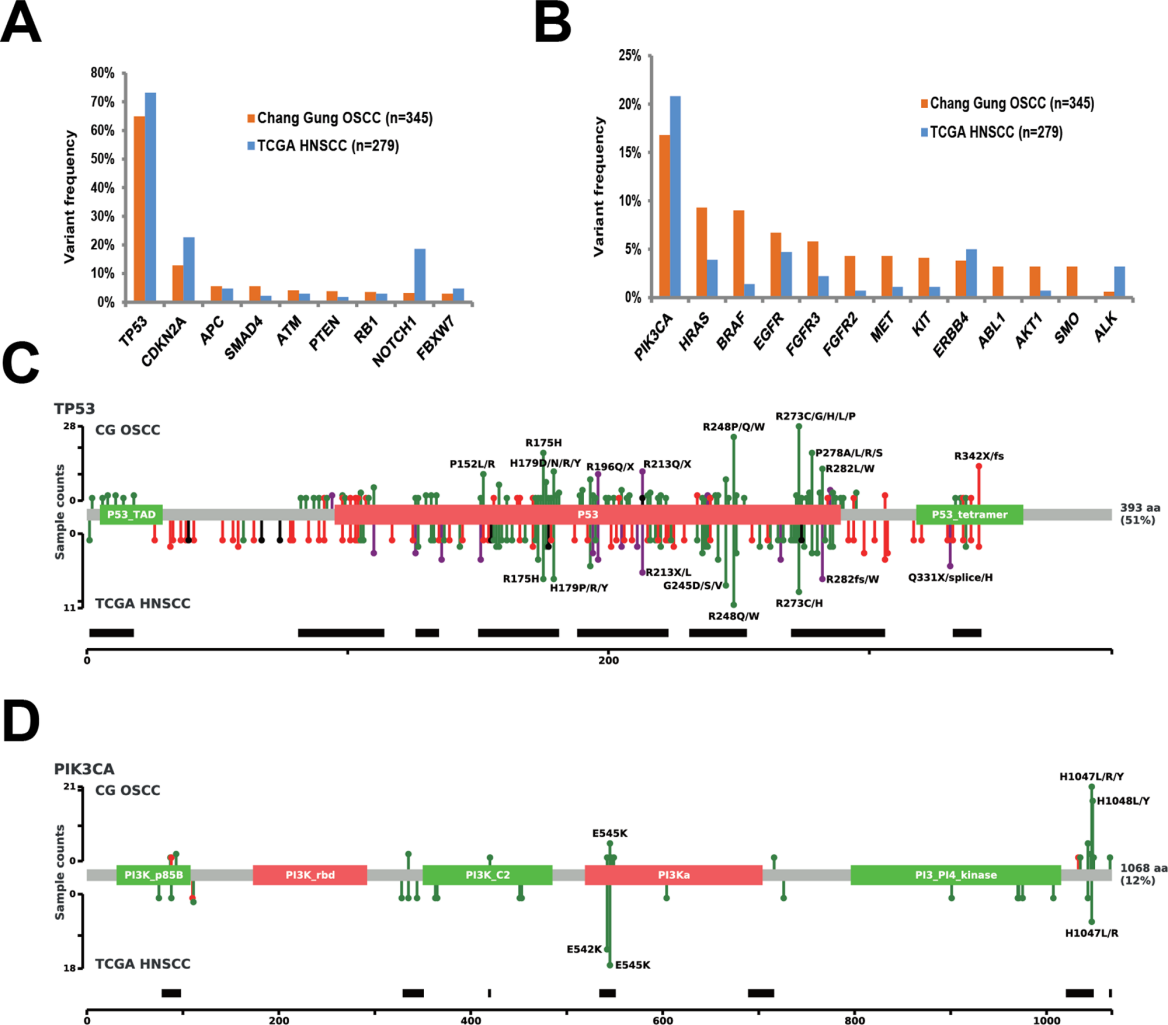
Extent of genetic disruption in advanced OSCC **A.** Prevalence of tumors harboring tumor suppressor gene variants in the Chang Gung cohort and in the TCGA HNSCC cohort. **B.** Prevalence of tumors harboring oncogenic variants in the Chang Gung cohort and in the TCGA HNSCC cohort. **C**. Distribution of *TP53* mutations in the Chang Gung cohort and in the TCGA HNSCC cohort. **D**. Distribution of *PIK3CA* mutations in the Chang Gung cohort and in the TCGA cohort.

The 1,269 non-synonymous mutations identified in the current study were located in 44 genes (Figure 2). The most frequently mutated genes were *TP53 (65%)*, *PIK3CA (16.8%), CDKN2A (12.8%), HRAS* (9.3%), *BRAF* (9.0%), *EGFR* (6.7%) and *FGFR3* (5.8%). Genetic mutations in the ten most frequently mutated genes were identified in 263 (76.2%) samples (Figure 2). As only the hotspot regions of 45 cancer-related genes were sequenced in our study, we analyzed whether our targeted sequencing approach could distort the observed mutation spectra. We therefore compared our findings with the mutational patterns reported in The Cancer Genome Atlas (TCGA) head and neck squamous cell carcinoma (HNSCC) dataset (containing the whole-exome sequencing data of 279 tumors). The frequency of genetic variations in TSG detected in our study was largely similar to that observed in TCGA dataset, the only exceptions being *CDKN2A* (12.8% vs. 22.6% in the TCGA data) and *NOTCH1* (3.2% vs. 18.6% in the TCGA data) which showed a significantly lower degree of sequence variation in our study (Figure 3A). In contrast, mutations in several oncogenes (including potential drug targets) were more commonly observed in our study than in the TCGA (Figure 3B). Notably, several oncogenes had a 3-fold higher mutation rates in the current report compared with the TCGA data, including *AKT1* (3.2% vs. 0.7%), *BRAF* (9% vs. 1.4%), *CTNNB1* (2.3% vs. 0.7%), *FGFR1* (1.4% vs. 0.4%), *FGFR2* (4.3% vs. 0.7%), *KIT* (4.1% vs. 1.1%), *KRAS* (2.3% vs. 0.4%), and *MET* (4.3% vs. 1.1%; Figure 3B). Moreover, we also detected 11 samples with *ABL1* (3.2%) mutations and 11 cases with *SMO* (3.2%) mutations. No such mutations were reported in the TCGA dataset. Mutations in the PI3K pathway, including *PIK3CA*, *AKT1* and *PTEN*, were identified in 68 (19.7%) tumors, suggesting that these patients may benefit from AKT-PI3K-mTOR inhibitors.

### Molecular characterization of the detected mutations

The four most commonly mutated genes were selected for further analysis. *TP53* showed sequence mutations in 224 (65%) samples (324 mutations distributed across 142 loci). The majority of *TP53* mutations were missense mutations (80.2%), followed by nonsense mutations (14.2%), and splice-site mutations (2.5%). The distribution of mutation types in our study is similar to the TCGA data. Most (67.6%) of the TP53 mutations were disruptive mutations (46 nonsense mutations, 7 indels, 8 splice-site mutations, and 158 missense mutations leading to changes in charged amino acids) [16]. We also identified 13 tumors harboring two mutations in *TP53*, likely due to the presence of multiple subclones in the sequenced specimens. Although our targeted region covers only 69% of the entire coding region, the mutational spectrum of TP53 observed in our study is highly similar to the *TP53* mutation spectrum observed in the TCGA HNSCC dataset (Figure 3C). PIK3CA was the most frequently mutated oncogene in our study, with a total of 65 genetic variants (63 missense and 2 nonsense mutations) identified in 58 (16.8%) tumors. Both of the two nonsense mutations were identified in tumors characterized by a hypermutator phenotype. The most common PIK3CA mutational hotspots detected in our study were located in exon 20 (H1047, G1049), whereas the most frequently mutated hotspots in the TCGA HNSCC study were located in the exon 9 (E542 and E545; Figure 3D). We also identified 53 *CDKN2A* mutations in 44 (12.8%) tumors; of them, 40 were definitely inactivating mutations (29 nonsense mutations, 3 indels, and 8 splice site mutations). The most commonly identified *CDKN2A* variant was the R58X nonsense mutations (identified in 20 tumors). According to the COSMIC database, the R58X mutation is frequently detected in cancers from upper aerodigestive trait, skin, esophagus, pancreas, and lung. *HRAS* mutations were identified in 32 tumors; all of them were well-known missense mutations located on three specific amino acid residues, G12 (*n* = 13), G13 (*n* = 9), and Q61 (*n* = 6). Notably, the allele-specific G12S mutation on *HRAS* was detected at a higher frequency (*n* = 9 in 345 subjects) in our cohort than in the TCGA HNSCC study (*n* = 2 in 279 subjects). The G12S mutation in *KRAS* has been previously associated with radiation resistance in cultured cell lines [17]. Overall, the frequency and type of mutations observed in the *TP53*, *CDKN2A*, *PIK3CA*, and *HRAS* genes were consistent with both previous genetic studies of OSCC as well as the TCGA HNSCC dataset [18–21]. The subtle difference in specific alleles may reflect difference in etiology, ethnicity, or tumor stage between the two cohorts.

### Genetic mutations associated with survival rates

**Figure 4:**
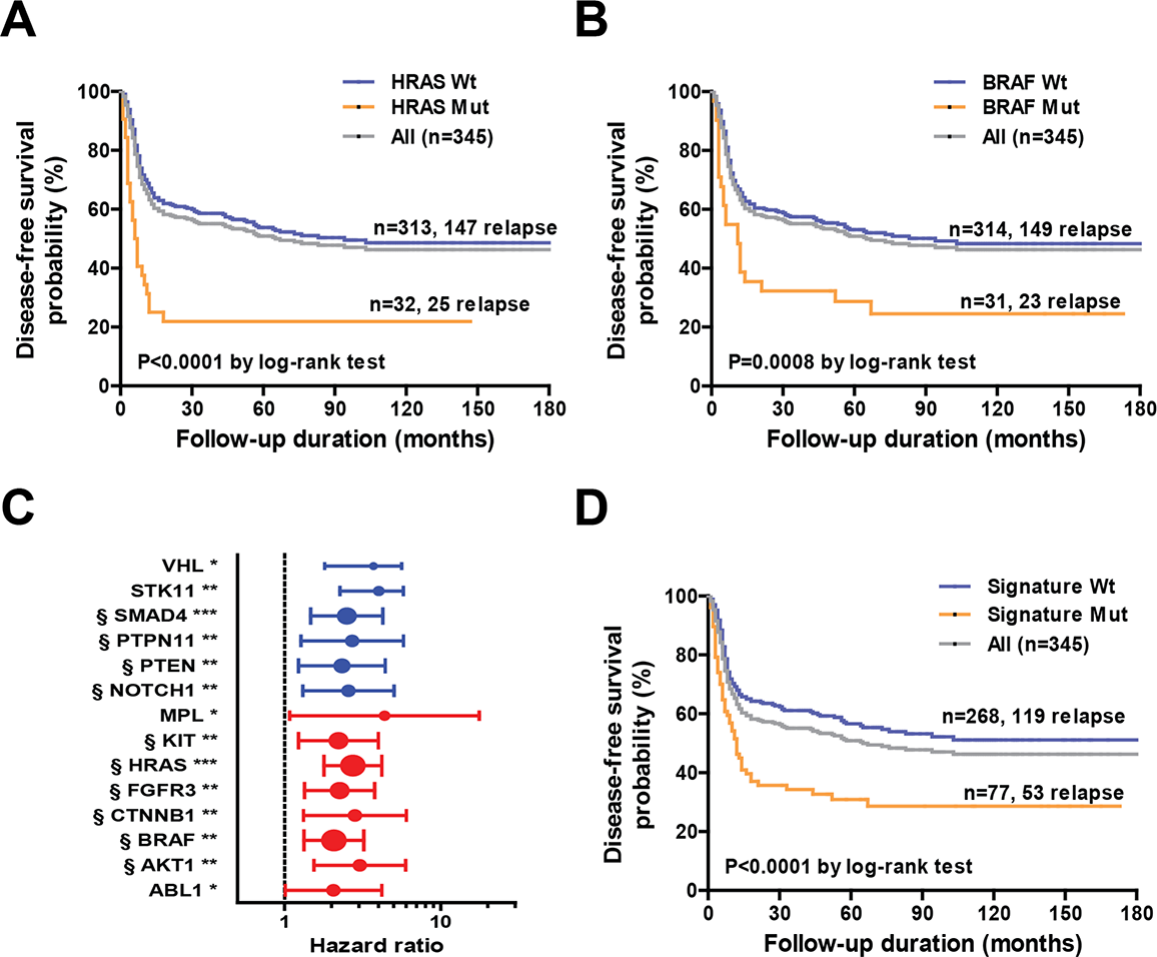
A. Kaplan-Meier analysis of disease-free survival in the entire cohort (*n* = 345) based on *HRAS* mutation status **B.** Kaplan-Meier analysis of disease-free survival in the entire cohort (*n* = 345) based on *BRAF* mutation status. **C.** Univariate hazard ratios and 95% confidence intervals for disease relapse according to specific genetic alterations in individual genes. The strength of statistical significance identified in Cox proportional hazard models is reported in brackets (***P < 0.001, **P < 0.01, *P < 0.05). § denotes genes selected in the genetic signature. **D.** Kaplan-Meier analysis of disease-free survival of the entire cohort (*n* = 345) according to the genetic signature mutation status.

We used Cox proportional hazard regression to identify the genetic variants independently associated with survival outcomes. Genetic mutations located in 14 genes (*ABL1, AKT1, BRAF, CTNNB1, FGFR3, HRAS, KIT, MPL, NOTCH1, PTEN, PTPN11, SMAD4, STK11*, and *VHL*) were significantly associated with DFS in univariate Cox regression analysis (Table 2); among them, mutations in 11 genes (*AKT1, BRAF, CTNNB1, FGFR3, HRAS, KIT, NOTCH1, PTEN, PTPN11, SMAD4* and *STK11*) were found to be high-risk factors for poorer survival (hazard ratio > 2 and *P* < 0.01; Table 2). Kaplan-Meier analyses confirmed the presence of statistically significant differences in terms of survival according to the mutational status of individual genes (Table 2), with two genes in the EGFR signaling pathway, *HRAS* and *BRAF*, showing the most significant difference. The median DFS for patients with and without *HRAS* mutation was 6.5 and 94 months (*p* < 0.0001) (Figure 4A). The median DFS rates for patients with and without *BRAF* mutations were 11 months and 94 months, respectively (*P* = 0.0008; Figure 4B). Other variables found to be significantly associated with DFS in the entire cohort were pT3-4 tumor status, pN2 nodal status, pathological stage IV, positive ECS status, close margins (≤ 4 mm), and treatment based solely on surgery (Table 2).

**Table 2:** Univariate analyses of the prognostic gene panel and traditional risk factors in relation to disease-free survival in 345 OSCC patients

Variable	N (%)	HR	95% CI	*p*-value	*q*-value^a^	*p*-value^b^	Marker^c^
Traditional risk factor							
Age (≥ 65 vs < 65 years)	38 (11.0)	0.88	0.54 – 1.46	0.626	0.227	0.620	
Gender (male vs female)	325 (94.2)	1.23	0.61 – 2.51	0.562	0.189	0.555	
Extracapsular spread (Y vs N)	201 (58.3)	1.90	1.38 – 2.61	< 0.001	< 0.001	< 0.001	
Stage (IV vs III)	260 (75.4)	2.26	1.50 – 3.41	< 0.001	< 0.001	< 0.001	
T-status (pT34 vs pT12)	192 (55.6)	1.91	1.39 – 2.61	< 0.001	< 0.001	< 0.001	
N-status (pN2 vs pN1)	222 (64.3)	2.04	1.45 – 2.88	< 0.001	< 0.001	< 0.001	
Margin (≤ 4 vs > 4 mm)	43 (12.5)	2.03	1.37 – 3.01	< 0.001	< 0.001	< 0.001	
Perineural invasion (Y vs N)	177 (51.3)	1.22	0.91 – 1.65	0.186	0.087	0.179	
Vascular invasion (Y vs N)	18 (5.2)	1.04	0.53 – 2.04	0.901	0.277	0.899	
Lymphatic invasion (Y vs N)	44 (12.7)	1.85	1.25 – 2.73	0.002	0.003	0.001	
Treatment (surgery only vs surgery plus RT/CCRT)	25 (7.2)	1.78	1.06 – 2.98	0.028	0.022	0.024	
Genotype (Mut vs Wt)							
*ABL1*	11 (3.2)	2.06	1.01 – 4.19	0.046	0.031	0.039	
*AKT1*	11 (3.2)	3.04	1.55 – 5.97	0.001	0.003	< 0.001	Y
*BRAF*	31 (9.0)	2.07	1.33 – 3.22	0.001	0.003	< 0.001	Y
*CTNNB1*	8 (2.3)	2.83	1.33 – 6.03	0.007	0.009	0.004	Y
*FGFR3*	15 (4.3)	2.25	1.34 – 3.77	0.002	0.004	0.001	Y
*HRAS*	32 (9.3)	2.74	1.79 – 4.19	< 0.001	< 0.001	< 0.001	Y
*KIT*	14 (4.1)	2.21	1.23 – 3.99	0.008	0.009	0.006	Y
*MPL*	2 (0.6)	4.37	1.08 – 17.67	0.039	0.003	0.021	
*NOTCH1*	11 (3.2)	2.57	1.31 – 5.03	0.006	0.009	0.004	Y
*PTEN*	13 (3.8)	2.33	1.23 – 4.42	0.009	0.009	0.007	Y
*PTPN11*	8 (2.3)	2.72	1.27 – 5.79	0.010	0.009	0.006	Y
*SMAD4*	19 (5.5)	2.50	1.47 – 4.26	0.001	0.002	< 0.001	Y
*STK11*	7 (2.0)	3.25	1.43 – 7.35	0.005	0.008	0.002	
*VHL*	4 (1.2)	2.74	1.01 – 7.40	0.047	0.031	0.035	

Abbreviations: HR, hazard ratio; CI, confidence interval; RT, radiotherapy; CCRT, concurrent chemoradiation.

a *q*-value calculated with the Storey method

b Kaplan-Meier anaysis, log-rank test *p*-value

c Selected in the gene signature

### Prognostic mutation-based biomarker panel

As patients harboring specific gene mutations represented only a small fraction of the entire cohort, we sought to generate a mutation-based prognostic signature through the combined evaluation of multiple genetic mutations. To this aim, we selected 10 statistically significant (*P* < 0.01; false discovery rate-adjusted *p*-value < 0.05) genes with adequate population frequency (>2%). A similar criterion was used to identify mutated genes significantly associated with survival by Kandoth et al. [22]. The mutation-based gene signature consisted of *HRAS, BRAF, FGFR3, SMAD4, KIT, PTEN, NOTCH1, AKT1, CTNNB1*, and *PTPN11* (Figure 4C). A patient was considered signature-positive if one or more genes in the signature panel were mutated. A total of 77 patients in our cohort harbored at least one mutation included in the gene signature. There were 53 (68.8%) relapse cases among the 77 patients with the mutated signatures. In contrast, only 119 (44.4%) relapsed cases were identified among the 268 patients with wild-type signatures. Kaplan-Meier curves confirmed the presence of statistically significant differences in terms of survival according to the gene signature status (Figure 4D). The median DFS periods for patients with and without the mutated signature were 12 months and > 180 months, respectively (*p* < 0.0001) (Figure 4D). The hazard ratio for disease relapse for patients with a mutated gene signature (compared with those harboring wild-type gene signatures) was 2.03 (95% confidence interval: 1.47−2.81, Table 3).

**Table 3:** Univariate analyses of the prognostic gene panel in relation to disease-free survival in the entire cohort and different patient subgroups

Group (N, event, %)	Signature mutant		Signature Wt		HR	95% CI	*p*-value	*q*-value^a^	***p***-value^b^
	N	Event (%)	N	Event (%)					
Entire cohort (345, 172, 49.9)	77	53 (68.8)	268	119 (44.4)	2.03	1.47–2.81	< 0.001	< 0.001	< 0.0001
Subgroup A (172, 93, 54.1)	43	31 (72.1)	129	62 (48.1)	1.96	1.27–3.02	0.002	0.031	0.0016
Subgroup B (173, 79, 45.7)	34	22 (64.7)	139	57 (41.0)	2.04	1.25–3.34	0.005	0.008	0.0033
1996–2003 (143, 83, 58)	38	27 (71.1)	105	56 (53.3)	1.73	1.09–2.75	0.020	0.168	0.0158
2004–2011 (202, 89, 44.1)	39	26 (66.7)	163	63 (38.7)	2.26	1.43–3.57	< 0.001	0.002	0.0003

Abbreviations: Wt, wild-type; HR, hazard ratio; CI, confidence interval.

a *q*-value calculated with the Storey method

b Kaplan-Meier anaysis, log-rank test *p*-value

### Subgroup validation of the prognostic gene signature

**Figure 5:**
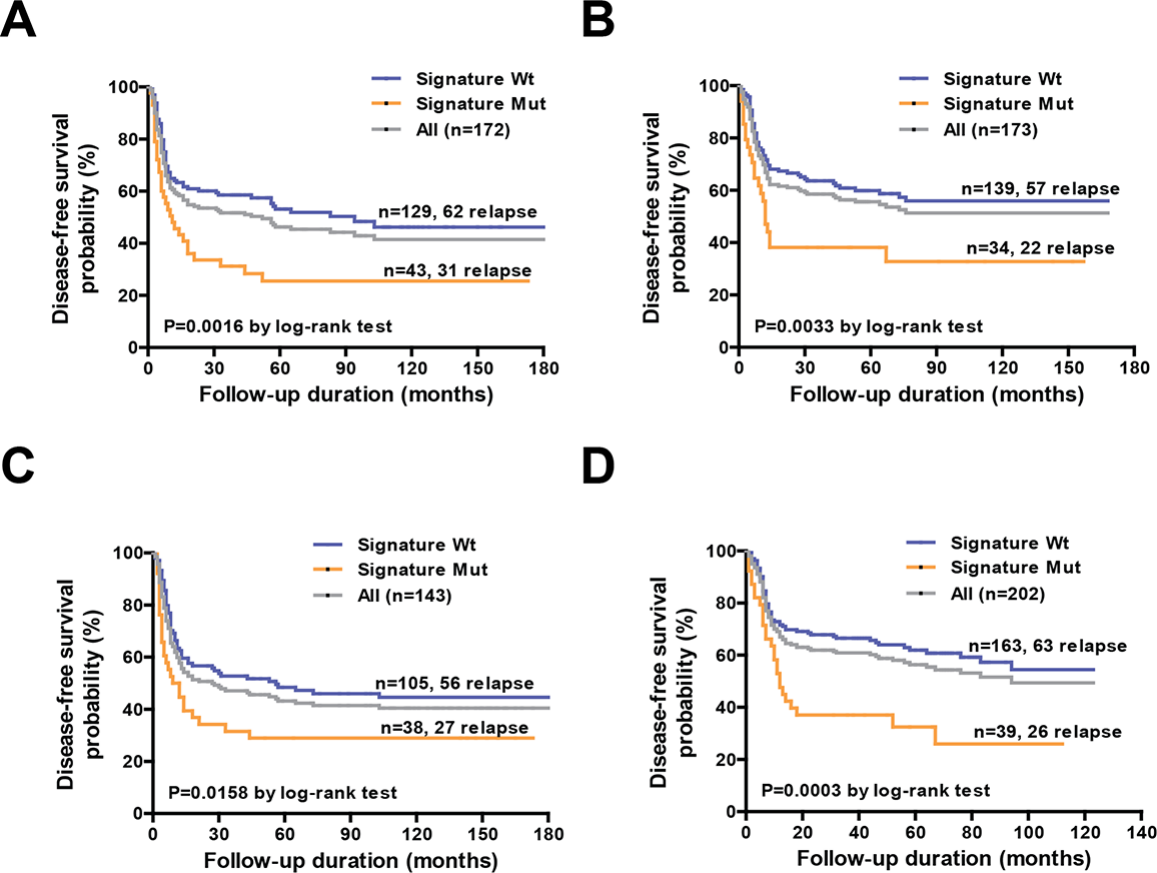
Association of the mutation-based genetic signature with disease-free survival in specific subgroups **A, B.** Kaplan-Meier analysis of disease-free survival in subgroup A (*n* = 172) and subgroup B (*n* = 173) according to the genetic signature mutational status. **C, D.** Kaplan-Meier analysis of disease-free survival in the year 1996–2003 cohort (*n* = 143) and year 2004–2011 cohort (*n* = 202) according to the genetic signature mutation status.

We further analyzed the reproducibility of the identified gene signature using two different resampling approaches. First, we randomly divided the cohort into two test subgroups − subgroup A (*n* = 172) and subgroup B (*n* = 173) − with a similar sample size and a comparable distribution of clinicopathological risk factors (Figure 1, Table 1). We identified 93 (54.1%) and 79 (45.7%) relapsed in subgroup A and subgroup B, respectively. We then estimated the hazard ratios associated with the presence of the prognostic gene signature for DFS in each subgroup using Cox regression. In both subgroups, patients with mutations in the gene signature showed significantly poorer DFS compared with wild-type patients (Table 3). The median DFS periods for patients with and without mutations in the gene signature for subgroup A and subgroup B were 11 vs. 94 months (*P* = 0.0016) and 12 vs. >180 months (*P* = 0.0033), respectively (Figures 5A, 5B). In the second resampling approach, we divided the entire study cohort into two different subgroups based on the time of patient enrollment. The 1996−2003 group consisted of 143 samples collected between 1996 and 2003, whereas the 2004−2011 group consisted of 202 samples collected between 2004 and 2011 (Figure 1, Table 1). In each group, we identified a total of 83 (58%) and 89 (44.1%) relapsed cases, respectively. We also analyzed the hazard ratios of the prognostic gene signature for DFS in each subgroup. In both subgroups, the prognostic gene signature was significantly associated with an increased risk of disease relapse (Table 3). Kaplan-Meier curves confirmed the presence of statistically significant differences in terms of DFS according to the prognostic gene signature in both subgroups (Table 3). The median DFS periods for patients with and without the prognostic gene signature in subgroup 1996–2003 and subgroup 2004–2011 were 10.5 vs. 56 months (*P* = 0.016) and 12 vs. >180 months (*P* = 0.0003), respectively (Figure 5C, 5D).

### Associations between the prognostic gene signature and standard prognostic factors

The associations between the prognostic gene signature and standard prognostic factors are illustrated in Table 4. The prognostic gene signature was significantly associated with the pathological T status (*P* = 0.038). Although pT1-2 tumors were signature-positive in only 17% of cases, 26.6% of all pT3-4 tumors were found to be positive. We did not identify other significant relationships between the prognostic gene signature and other known prognostic factors, including age, sex, alcohol, betel nut chewing, cigarette smoking, pathological N status, pathological staging, and ECS status (Table 4).

**Table 4:** Association between the prognostic gene signature and the general characteristics of the 345 OSCC patients

Variable	Signature mutant	Signature wild-type	*p*-value
N (%)	77 (22.3)	268 (77.7)	
Age at onset (years)			0.409
< 65	71 (23.1)	236 (76.9)	
≥65	6 (15.8)	32 (84.2)	
Sex			1.0
Male	73 (22.5)	252 (77.5)	
Female	4 (20.0)	16 (80.0)	
Alcohol drinking			0.777
No	23 (23.2)	76 (76.8)	
Yes	54 (22.0)	192 (78.0)	
Betel quid chewing			1.0
No	14 (22.2)	49 (77.8)	
Yes	63 (22.3)	219 (77.7)	
Cigarette smoking			0.263
No	10 (31.3)	22 (68.7)	
Yes	67 (21.4)	246 (78.6)	
Pathological T status			0.038
pT1-2	26 (17.0)	127 (83.0)	
pT3-4	51 (26.6)	141 (73.4)	
Pathological N status			0.105
pN1	21 (17.1)	102 (82.9)	
pN2	56 (25.2)	166 (74.8)	
Pathological stage			0.294
III	15 (17.7)	70 (82.3)	
IV	62 (23.9)	198 (76.1)	
Extracapsular spread			0.794
No	31 (21.5)	113 (78.5)	
Yes	46 (22.9)	155 (77.1)	
Perineural invasion			0.898
No	38 (22.6)	130 (77.4)	
Yes	39 (22.0)	138 (78.0)	
Lymphatic invasion			0.698
No	66 (21.9)	235 (78.1)	
Yes	11 (25.0)	33 (75.0)	
Vascular invasion			0.383
No	75 (22.9)	252 (77.1)	
Yes	2 (11.1)	16 (88.9)	
Margin status^a^			0.333
≤ 4 mm	12 (27.9)	31 (72.1)	
> 4 mm	64 (21.5)	234 (78.5)	
Treatment modality			0.004
Surgery only	12 (48)	13 (52)	
Surgery + RT/CCRT	65 (20)	255 (80)	
Relapse status			< 0.001
No	24 (13.9)	149 (86.1)	
Yes	53 (30.8)	119 (69.2)	

Abbreviations: RT, radiotherapy; CCRT, concurrent chemoradiation.

a Results available for 341 patients.

### Multivariate analysis

In the entire study cohort, the 5-year DFS was 51%. We then examined the entire study cohort (*n* = 345) with respect to the ability of the prognostic gene signature and other clinicopathological risk factors (pT3–4 vs. pT1–2, pN2 vs. pN1, p-Stage IV vs. p-Stage III, ECS [yes vs. no], perineural invasion [yes vs. no], lymphatic invasion [yes vs. no], vascular invasion [yes vs. no], margin status [positive margins/close margins vs. clear margins, i.e. ≤ 4 mm vs. > 4 mm], surgery alone vs. surgery plus RT or CCRT) to predict DFS. Table 5 shows the results of multivariate analyses of 5-year DFS in the entire study cohort. The presence of ECS (*P* = 0.021), pT3–4 disease (*P* = 0.001), pN2 (*P* = 0.005), close margins (*P* = 0.01), and surgery alone (*P* < 0.001) were independent risk factors for DFS. The hazard ratio for disease relapse for patients with a mutated gene signature (compared with those harboring wild-type gene signatures) was 1.62 (95% confidence interval = 1.16−2.28, *P* = 0.005, Table 5). These results indicated that the prognostic gene signature was independently associated with the DFS even after allowance for traditional risk factors.

**Table 5:** Multivariate analyses of the prognostic gene panel and traditional risk factors in relation to disease-free survival in 345 OSCC patients

Variable	N	HR	95% CI	*p*-value
Mutation signature (Mutant vs Wild type)	77	1.624	1.157 – 2.279	0.005
Extracapsular spread (Yes vs No)	201	1.537	1.066 – 2.216	0.021
T-status (pT34 vs pT12)	192	1.750	1.260 – 2.431	0.001
N-status (pN2 vs pN1)	222	1.775	1.194 – 2.638	0.005
Stage (IV vs III)	260	ns	-	< 0.001^a^
Margin (≤ 4 vs > 4 mm)	43	1.707	1.139 – 2.559	0.010
Treatment (surgery only vs surgery plus RT/CCRT)	25	3.127	1.772 – 5.516	< 0.001

Abbreviation: HR, hazard ratio; RT, radiotherapy; CCRT, concurrent chemoradiation; CI, confidence interval; ns, not significant.

a *p*-value obtained in univariate analyses

## DISCUSSION

Previous whole-exome sequencing studies of tumor specimens have shed more light on the mutation landscape of OSCC and identified several novel pathogenic mutations. However, due to limitations in sequencing depth, such reports have often failed to identify low-frequency mutations located in known causative genes [10–12]. Moreover, previous studies lacked long-term survival data useful to investigate the clinical impact of such mutations [10–12]. This study specifically aimed at detecting actionable mutations for targeted therapy and investigating the prognostic significance of genetic mutations in a large series of OSCC samples. To this aim, we used ultra-deep NGS to target hotspot regions of genes that may act as oncogenic drivers and/or serve as drug targets.

Our study yielded at least four important results. First, we demonstrated that FFPE samples are suitable for ultra-deep NGS and that storage time of tumor specimens does not have affect sequencing results [23]. The ability to uncover genetic alteration in well-annotated FFPE samples thus allows studying the prognostic significance of common genetic alterations even under a retrospective study design. Second, our ultra-deep NGS approach had a high sensitivity for the detection of cancer-related mutations [24]. The high sensitivity of ultra-deep NGS can be attributed to its deeper coverage (> 2400-fold) compared with whole-exome sequencing. Third, at least 3% of our samples harbored sequence variants in genes that were not previously implicated in OSCC pathogenesis, including *BRAF*, *ABL1*, *AKT1*, *KIT*, *MET*, *FGFR2*, and *FGFR3* [25, 26]. Notably, therapeutic agents targeting such genes are currently available or under development [27–29]. Finally, we identified a prognostic gene signature that predicts both DFS and OS.

Although we specifically focused on target regions with known mutation hotspots, our findings indicate that ultra-deep NGS allows a reliable broad mutation testing for most oncogenes and tumor suppressor genes. For example, while we only covered 68% of the *TP53* coding region (including most of the DNA-binding region), the mutation frequency and spectrum of *TP53* observed in our study were in line with those reported in the TCGA HNSCC dataset [10]. Although the number of genetic variations observed in *NOTCH1* was significantly lower in our study [10, 11], this may be caused by the limited targeted sequence of *NOTCH1* (that covered only 2.1% of the entire transcript). Preliminary sequencing data covering the entire *NOTCH1* coding region revealed a mutation frequency similar to that reported in TCGA (data not shown).

Although the tumor proportion varied from 15 to 90% and tissue microdissection was not performed, we were able to confirm >90% of the identified mutations by orthogonal sequencing techniques. Oncogenic mutations are typically heterozygous and can sometimes co-occur with amplification of the wild-type allele in the same tumor, which can further complicate their detection in tumor specimens [30]. In order to overcome these issues, sequencing depth should be increased as much as possible [24]. Using an average depth greater than 2000×, herein we were able to confidently identify mutations with an allele frequency as low as 3% in a single sample. We detected 12 frequently mutated oncogenes (frequency ≥ 3%) in this cohort. Compared to the TCGA HNSCC data, 8 oncogenes were observed at 3-fold higher population frequency and 2 oncogenes were detected only in our study. Taken together, these findings suggest that ultra-deep NGS of hotspot regions is a highly sensitive method for detecting mutations in actionable cancer-related genes.

Slight differences in the mutation landscape were evident between the current study and the TCGA HNSCC dataset. For example, herein we detected the *HRAS* p.G12S allele in 9 cases (2.6%) compared with 2 cases (0.7%) in the TCGA data. The germline p.G12S *HRAS* mutation has been causally linked to Costello syndrome, a complex developmental disorder characterizing by facial dysmorphism [31, 32]. Moreover, the p.G12S *HRAS* somatic mutation has been mainly observed in upper aerodigestive tract malignancies [33]. Similar differences are evident for *PIK3CA* mutations. We observed 45 cases (13%) with mutations in the kinase domain (M1043, A1046, H1047, H1048, and G1049 mutations on exon 20) and 7 cases (2%) with mutations in the helical domain (E542, E545, and D549 mutations on exon 9). In contrast, the TCGA HNSCC dataset identified only 10 cases (3.6%) with kinase domain mutations (M1043 and H1047 on exon 20) and 32 cases (11.5%) with helical domain mutations (E542 and E545 on exon 9). Such differences in mutational hotspots can be attributed to differences in exposure to known carcinogens, disease stages, ethnicity, or genetic background. Previous studies have shown that helical domain mutations (e.g., p.E545K) activate the pathway by disrupting the p85-mediated inhibition [34], whereas kinase domain mutations (e.g., p.H1047R) promote an association with lipids to enhance the kinase activity [34]. A recent meta-analysis reported that *PIK3CA* exon 20 mutations are associated with a significantly shorter DFS in *KRAS* wild-type metastatic colorectal cancer patients treated with anti-EGFR antibody cetuximab [35]. A total of 68 cases (19%) in this study were found to harbor mutations in genes involved in the AKT-PI3K-mTOR pathway, suggesting that its specific inhibitors may warrant further studies in advanced OSCC patients.

In summary, ultra-deep NGS was clinically useful for identifying specific gene variants predicting prognosis in patients with advanced OSCC. The identified genetic panel needs independent validation before being introduced in clinical practice.

## METHODS

### Tumor samples

We retrospectively analyzed tumor samples from 345 nodal-positive, stage III/IV OSCC patients who were treated in Chang Gung Memorial Hospital between 1996 and 2011. The patients had not been previously treated, had no proven metastatic disease at the time of diagnosis and no synchronous or metachronous-occurring cancer. The primary inclusion criterion was amplifiable DNA from FFPE tissue. All patients were followed at least 30 months or until death. The estimated median follow-up time (calculated by the reverse Kaplan-Meier method) was 107 months. The pathological diagnosis of each OSCC case was confirmed using hematoxylin-eosin staining. Patients were staged according to the 2010 (7th) American Joint Committee on Cancer (AJCC) staging criteria. All of the study patients were treated by surgery, radiotherapy, or combined chemoradiotherapy at the Chang Gung Memorial Hospital between 1996 and 2011. Primary tumors were excised with safety margins of 1 cm or greater. Patients with cN+ disease received level I-V neck dissection, whereas patients with cN- disease received level I-III neck dissection. Post-operative radiotherapy (60 Gy) was given to patients with pathological risk factors according to the NCCN guidelines (before 2008) or the Chang Gung guidelines (as of 2008). Concomitant chemoradiation (CCRT) with cisplatin-based regimens were administered to patients with ECS, multiple lymph node metastases, or positive margins. Because the entire cohort was considered as a single test-set, positive findings required validated in an independent patient population. The number of patients, frequencies of sequences, and the study power depended on individual genetic mutations. Under the assumption of a 5-year DFS rate of 50% for patients with a positive mutation signature, a sample size of 345 patients will have 87% power at a significance level of 5% to detect an absolute difference of 15% (e.g. 42.5% vs 57.5%) in DFS associated with a positive signature. The study protocol followed the tenets of the Helsinki declaration and was approved by the Institutional Review Board of Chang Gung Memorial Hospital (CGMH 101-4457B). Patient consent was waived due to the retrospective nature of the study.

### Sample preparation, DNA sequencing and data processing

Tumor specimens were obtained at the time of surgery and fixed with formalin following standard protocols. Archived FFPE blocks were stored at ambient temperature for up to 16 years. All samples used for DNA extraction contained a minimum of 15% DNA derived from tumor cells. For each tumor, one roll of ten-micrometer FFPE sections was used for genomic DNA extraction and library preparation. Of the 355 independent tumors, 345 tumors had amplifiable DNA. Targeted regions of 45 cancer-related genes (Data Supplementary Table S1 and Table S2) were amplified by PCR, barcoded, and sequenced using the Ion Torrent PGM system with the Ion 318 chip. Raw reads were mapped to the hg19 reference genome using Torrent Suite Server (version 3.2) and variants were identified using the Torrent Variant Caller plug-in (version 3.2). Common variants in dbSNP 138 and 1000 Genome project were filtered out. The remaining genetic variants were annotated using the ANNOVAR [36] and CPAP [37] pipelines. Selected mutations were confirmed using Sanger sequencing or pyrosequencing. Details regarding sequencing and data processing are provided in the Data Supplement. All sequencing and variant annotation were performed by the Genomic Core (HL) and the Bioinformatics Core (PJH) of the Molecular Medicine Research Center at Chang Gung University without knowledge of the clinical data.

### Statistical analysis

Differences between the study groups were analyzed using Student's *t*-tests or Fisher's exact tests, as appropriate. Samples classified as unknown for any variable were excluded. The primary endpoint, DFS, was defined as the period of time in months from the date of surgery to the date of local or distant progression, death from any causes, or the date of last follow-up. Relative risk of relapse associated with mutations in the 45 genes tested were estimated from univariate Cox proportional hazards model for the entire cohort. Multiple test analyses was adjusted by using the *q*-value method to control for the false discovery rate. All genes with an adequate population frequency (> 2%) associated with DFS in the univariate analysis with *p* < 0.01 and *q*-value < 0.05 were selected to construct a mutation-based gene signature. A similar criterion was used to identify mutated genes significantly associated with survival by Kandoth et al. [22]. The mutation-based gene signature involved the *HRAS, BRAF, FGFR3, SMAD4, KIT*, *PTEN, NOTCH1, AKT1, CTNNB1,* and *PTPN11* genes. A patient was considered signature-positive if one or more genes in the signature panel were mutated. The prognostic significance of the gene signature was investigated using univariate Cox regression model in the entire cohort and each subgroup. To identify additional factors associated with DFS, we evaluated the following clinicopathological variables in a univariate Cox regression model: age (≥ 65 vs. < 65 years), sex (male vs. female), tumor status (pT3–4 vs pT1–2), nodal status (pN2 v pN1), AJCC stage (p-Stage IV vs. p-Stage III), ECS status (positive vs. negative), perineural invasion status (positive vs. negative), lymphatic invasion status (positive vs negative), vascular invasion status (positive vs negative), margin status (≤ 4 mm vs > 4 mm), treatment (only surgery vs. surgery plus RT or CCRT). All variables associated with DFS with *p* < 0.05 in univariate analysis and the gene signature mutation status were entered into a multivariate Cox regression models with a forced entry (or backward elimination) method. Disease-free survival in relation to gene signature mutation status was assessed with Kaplan-Meier survival curves (log-rank test). All analyses were performed using IBM SPSS Statistics v17. A two-tailed *p*-value < 0.05 was considered statistically significant.

## SUPPLEMENTAY FIGURES AND TABLES








