# Supplementary material for: Ultra-deep targeted sequencing of advanced oral squamous cell carcinoma identifies a mutation-based prognostic gene signature
Source: Oncotarget. 2015 Apr 25;6(20):18066–80. doi: 10.18632/oncotarget.3768 (PMC4621868; doi:10.18632/oncotarget.3768)
Supplement: Supplementary file 1 [file oncotarget-06-18066-s001.pdf]

SUPPLEMENTARY DATA

SUPPLEMENTARY METHODS

Ultra-deep targeted sequencing (UDT-Seq)

Genomic DNA was extracted from formalin-fixed paraffin-embedded (FFPE) tumor samples using the QIAmp DNA FFPE DNA extraction kit (Qiagen, Hilden, Germany). One non-surface FFPE section of 10-µm thickness was used for each tumor sample. DNA concentration was measured using both a NanoDrop spectrophotometer (Thermo Fisher Scientific, Wilmington, DE, USA) and a Qubit fluorometer (Invitrogen, Carlsbad, CA, USA). DNA integrity was assessed using real-time PCR. All of the PCR reactions were performed with approximately 20 ng of isolated genomic DNA using a GeneAmp® PCR System 9700 (Applied Biosystems, Foster City, CA, USA). The genomic regions of interest were targeted using the AmpliSeq™ Cancer Panel (version 1.0; Life Technologies, Carlsbad, CA, USA) containing a total of 189 primer pairs covering the selected regions of 45 cancer-related genes (29 oncogenes and 16 tumor suppressor genes) with well-documented roles in carcinogenesis (Data Supplementary Table S1). Amplified PCR products were ligated to barcode adapters and then subjected to five amplification cycles. Barcoded libraries were generated with the Ion Plus Fragment Library Kit (Life Technologies) using 50 ng of amplicons. Adaptor ligation, nick repair, and amplification were performed according to the Ion Torrent protocol (Life Technologies). The Agilent 2100 Bioanalyzer and the associated High Sensitivity DNA kit (Agilent Technologies) were used to determine the quality and concentrations of the libraries. Emulsion PCR and enrichment steps were carried out using the Ion OneTouch™ Template Kit (Life Technologies) according to the manufacturer's protocol. Sequencing of the amplicon libraries was carried out using the Ion Torrent PGM system with the Ion 318 chip. Barcoding was then performed using the Ion Xpress™ Barcode Adapters 1-16 Kit (Life Technologies). The Ion Sequencing Kit v.2 was used for all sequencing reactions according to the manufacturer's instructions.

Data Processing

Raw reads generated by sequencing were mapped to the hg19 reference genome using the Ion Torrent Suite (v. 3.2). Coverage depth was calculated using Torrent Coverage Analysis plug-in. Single nucleotide variants (SNVs) and short insertion/deletions (INDELs) were identified using the Torrent Variant Caller (version 3.2). To assess the false positive mutation rates resulted from technical issues such as homopolymers, mispriming and PCR artefacts, we sequenced the hotspot regions of the 45 genes using genomic DNA prepared from peripheral blood mononuclear cells (PBMC) from 16 different healthy subjects. With an average sequencing depth of >2000×, the mean error rate for the entire hotspot region was estimated at 0.3%. The mean error rate of individual nucleotide sequences is negatively correlated to the read depth. Therefore, we implemented a coverage-dependent threshold strategy to improve the sensitivity and to preserve the specificity of variant detection. The variant calling threshold was set at 3%, 5%, and 10% for variants with ≥ 1000×, 200-1000×, and < 200×, respectively. Furthermore, to reduce the rate of false positive mutations that could have arisen during FFPE samples storage, we selected a highly stringent strand bias threshold during the variant calling process using the Torrent Variant Caller (version 3.2). Based on the threshold setting, we identified 3,634 variants from the 345 sequenced samples. Common variants (MAF ≥ 1%) registered in dbSNP 138 (http://www.ncbi.nlm.nih.gov/projects/SNP) without a flag for clinical significance were filtered out. Variants with MAF ≥ 1% in 1000 Genome project were also filtered out. All of the remaining genetic variants were annotated using the ANNOVAR[1] and CPAP[2] pipelines. The PolyPhen-2 (http://genetics.bwh.harvard.edu/pph2/), SIFT (http://sift.jcvi.org/www/SIFT_BLink_submit.html), and Mutation-Taster (http://www.mutationtaster.org/) tools were used to evaluate the detected SNVs in terms of sequence conservation, chemical change, and probability of pathogenicity. We used the Catalogue Of Somatic Mutation In Cancer (COSMIC) database (release 61) (http://cancer.sanger.ac.uk/cancergenome/projects/cosmic/) to establish whether a variant was newly identified or previously reported. Selected variants were confirmed either by Sanger Sequencing using an ABI 3500xL Genetic Analyzer (Life Technologies) or using pyrosequencing on a PyroMark Q24 machine (Qiagen) according to the manufacturer's protocol.

Sequencing results

We were able to achieve a 2410-fold mean sequence coverage for the targeted regions, with 97.53% of them covered at > 100 folds. The sequences yielded an average of 517,843 ± 205,743 mapped reads for each sample; notably, 95 ± 1 % of the reads and 86 ± 2% of the bases were mapped to the targeted regions. The total coverage for all of the targeted bases was 100 ± 0% at 1×, 100 ± 1% at 20×, and 97 ± 2% at 100×, with a mean read depth of 2410 ± 962 (Data Supplementary Table S2). Based on a coverage-

dependent threshold setting, 3,634 variants with an allelic frequency ≥ 3% were detected. Among them, 1,973 variants had an entry in the dbSNP or 1000 Genome databases but no entry in the COSMIC database. These variants were annotated as SNP and disregarded for further analysis. The remaining 1,661 variants (detected in 285 samples) were considered as somatic mutations and were analyzed further.

REFERENCES

1. Wang K, Li M, Hakonarson H. ANNOVAR: functional annotation of genetic variants from high-throughput sequencing data. Nucleic Acids Res 38. 2010, e164.
2. Huang PJ, Yeh YM, Gan RC, et al. CPAP: Cancer Panel Analysis Pipeline. Hum Mutat. 2013; 34:1340–6.

SUPPLEMENTARY FIGURES AND TABLES

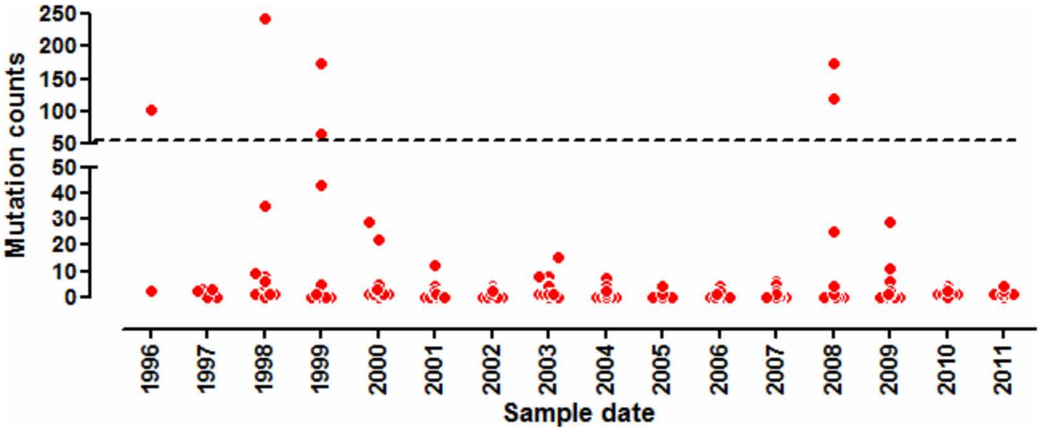

Supplementary Figure S1: Frequency of genetic variants detected in human OSCC specimens (*n* = 345).

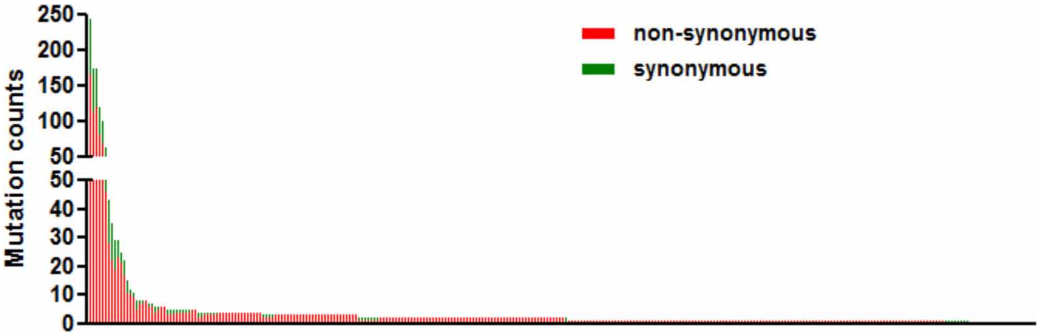

Supplementary Figure S2: Number of silent and non-silent variants detected in the targeted regions of the 276 samples which carried sequence variations.

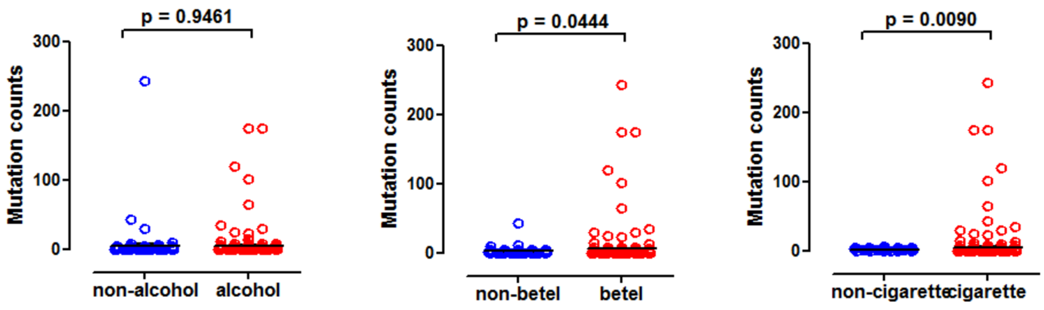

Supplementary Figure S3: Effect of alcohol drinking, betel quid chewing, and cigarette smoking on the genetic variants detected in human OSCC specimens (*n* = 345).

Supplementary Table S1: Targeted genes

Gene Symbol	Gene Type	RefSeq ID	Transcript length (nt)	UniPro ID	Protein length (aa)	Amplicon No	Covered region (nt)	Covered fraction (%)
ABL1	Onc	X16416	5766	P00519	1130	5	322	5.6
AKT1	Onc	ENST00000349310	2866	P31749	480	1	96	3.3
ALK	Onc	NM_004304	6220	Q9UM73	1620	2	107	1.7
APC	TSG	NM_000038	10701	P25054	2843	7	525	4.9
ATM	TSG	NM_000051	13147	Q13315	3056	17	994	7.6
BRAF	Onc	NM_004333	2480	P15056	766	2	212	8.5
CDH1	TSG	NM_004360.2	4875	Q14646	882	3	195	4.0
CDKN2A	TSG	NM_000077	218	P42771	156	1	88	40.4
CSF1R	Onc	NM_005211	3989	P07333	972	2	125	3.1
CTNNB1	Onc	NM_001904	3729	P35222	781	1	98	2.6
EGFR	Onc	NM_005228	9821	P00533	1210	8	724	7.4
ERBB2	Onc	NM_004448	4458	P04626	1255	4	276	6.2
ERBB4	Onc	NM_005235	12132	Q15303	1308	8	510	4.2
FBXW7	TSG	NM_033632.1	3896	Q969H0	707	5	305	7.8
FGFR1	Onc	NM_000604	5702	P11362	820	2	117	2.1
FGFR2	Onc	NM_000141.2	4255	P21802	821	4	281	6.6
FGFR3	Onc	NM_000142	4287	P22607	806	5	428	10.0
FLT3	Onc	Z26652	3848	P36888	996	5	299	7.8
GNAS	Onc	NM_000516.3	3480	P63092	1037	1	68	2.0
HNF1A	TSG	NM_000545.3	3468	P20823	850	2	146	4.2
HRAS	Onc	NM_005343	894	P01112	189	2	145	16.2
IDH1	Onc	NM_005896.2	2382	O75874	414	1	48	2.0
JAK3	Onc	NM_000215	5432	P52333	1124	2	129	2.4
KDR	Onc	NM_002253	5831	P35968	1356	9	563	9.7
KIT	Onc	NM_000222	5186	P10721	976	9	617	11.9
KRAS	Onc	NM_004985	5765	P01116	188	3	217	3.8
MET	Onc	NM_000245	6635	P08581	1390	5	348	5.2
MLH1	TSG	NM_000249.2	2752	P40692	756	1	58	2.1
MPL	Onc	NM_005373.1	1950	P40238	635	1	88	4.5
NOTCH1	TSG	NM_017617.2	9371	P46531	2555	2	195	2.1
NPM1	TSG	NM_002520.4	1758	P06748	294	1	97	5.5
NRAS	Onc	NM_002524	4449	P01111	189	2	98	2.2
PDGFRA	Onc	NM_006206	6576	P16234	1089	4	296	4.5

(*Continued*)

Gene Symbol	Gene Type	RefSeq ID	Transcript length (nt)	UniPro ID	Protein length (aa)	Amplicon No	Covered region (nt)	Covered fraction (%)
PIK3CA	Onc	NM_006218.1	9093	P42336	1068	7	464	5.1
PTEN	TSG	NM_000314.4	9027	P60484	403	7	479	5.3
PTPN11	TSG	NM_002834.3	6101	Q06124	593	2	146	2.4
RB1	TSG	NM_000321	4840	P06400	928	8	576	11.9
RET	Onc	NM_020975	5659	O43519	1114	5	339	6.0
SMAD4	TSG	NM_005359.3	8769	Q13485	552	8	548	6.2
SMARCB1	TSG	NM_003073.2	1728	Q12824	394	4	249	14.4
SMO	Onc	NM_005631.3	3738	Q99835	787	5	379	10.1
SRC	Onc	NM_005417	4631	P12931	536	1	59	1.3
STK11	TSG	NM_000455	3328	Q15831	433	4	362	10.9
TP53	TSG	NM_000546	2579	P04637	393	8	691	26.8
VHL	Onc	NM_000551.2	3737	Q9NWT6	213	3	204	5.5

Supplementary Table S2: Targeted regions

Supplementary Table S3: Sequencing coverage

Supplementary Table S4: Validation of PGM data with orthogonal sequencing technology
